# Evidence for Steric Regulation of Fibrinogen Binding to *Staphylococcus aureus* Fibronectin-binding Protein A (FnBPA)*

**DOI:** 10.1074/jbc.M113.543546

**Published:** 2014-03-13

**Authors:** Vaclav Stemberk, Richard P. O. Jones, Olga Moroz, Kate E. Atkin, Andrew M. Edwards, Johan P. Turkenburg, Andrew P. Leech, Ruth C. Massey, Jennifer R. Potts

**Affiliations:** From the Departments of ‡ Biology and; § Chemistry, University of York, York YO10 5DD, United Kingdom and; the ¶ Department of Biology and Biochemistry, University of Bath, Bath BA2 7AY, United Kingdom

**Keywords:** Fibrinogen, Fibronectin, Isothermal Titration Calorimetry, Staphylococcus aureus, Surface Plasmon Resonance (SPR), X-ray Crystallography, FnBPA

## Abstract

The adjacent fibrinogen (Fg)- and fibronectin (Fn)-binding sites on Fn-binding protein A (FnBPA), a cell surface protein from *Staphylococcus aureus*, are implicated in the initiation and persistence of infection. FnBPA contains a single Fg-binding site (that also binds elastin) and multiple Fn-binding sites. Here, we solved the structure of the N2N3 domains containing the Fg-binding site of FnBPA in the apo form and in complex with a Fg peptide. The Fg binding mechanism is similar to that of homologous bacterial proteins but without the requirement for “latch” strand residues. We show that the Fg-binding sites and the most N-terminal Fn-binding sites are nonoverlapping but in close proximity. Although Fg and a subdomain of Fn can form a ternary complex on an FnBPA protein construct containing a Fg-binding site and single Fn-binding site, binding of intact Fn appears to inhibit Fg binding, suggesting steric regulation. Given the concentrations of Fn and Fg in the plasma, this mechanism might result in targeting of *S. aureus* to fibrin-rich thrombi or elastin-rich tissues.

## Teaser

**Background:** *Staphylococcus aureus* fibronectin-binding protein A (FnBPA) binds fibronectin and fibrinogen at adjacent sites.

**Results:** The fibrinogen-binding mechanism is similar but not identical to homologous bacterial proteins. Ternary complex formation by intact fibronectin and fibrinogen on adjacent FnBPA sites could not be demonstrated.

**Conclusion:** Fibrinogen binding is sterically regulated by fibronectin binding.

**Significance:** Steric regulation might result in targeting of *S. aureus* to fibrin clots.

## Introduction

**FIGURE 1.**
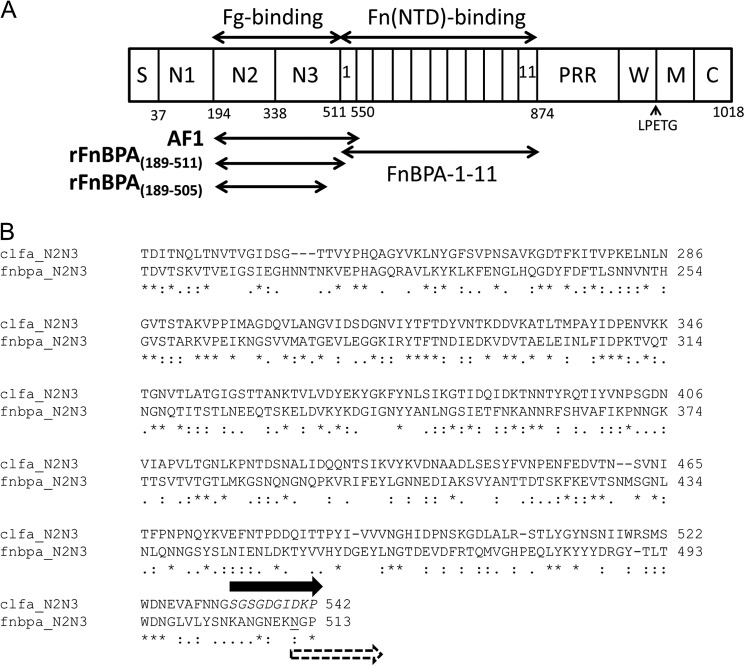
**Domain composition of *S. aureus* FnBPA and alignment of its N2N3 amino acid sequence with ClfA, also from *S. aureus*.** *A*, the domain composition of FnBPA. Described from the N terminus, FnBPA contains a secretion signal (*S*) followed by the N1-N3 region and the FnBRs FnBPA-1–11. N2N3 binds fibrinogen, and the FnBRs bind the NTD of Fn. The C-terminal region contains proline-rich repeats (*PRR*), cell wall (*W*), and membrane (*M*) spanning regions, a sortase A recognition motif (LPETG), and a short cytoplasmic region (*C*). Recombinantly expressed constructs used in this study are shown: rFnBPA_(189–505)_, rFnBPA_(189–511)_, and AF1 (which contains rFnBPA_(189–511)_ and FnBPA-1). *B*, alignment (ClustalW2, EMBL-EBI) of the N2N3 amino acid sequences from FnBPA (*lower rows*) and ClfA (*upper rows*). The ClfA latch strand residues are shown in *italics*, and the most N-terminal residue of FnBPA-1 involved in the interaction with the NTD from Fn is *underlined* (33). The potential overlap between residues affected by Fg (*solid arrow*) and Fn (*dashed arrow*) binding is highlighted by the *arrows*.

*Staphylococcus aureus* is a Gram-positive bacterium that can cause a variety of infections ranging from superficial skin infections to severe invasive infections, such as infective endocarditis (1); the emergence of antibiotic-resistant strains (2) presents significant therapeutic challenges. Infective endocarditis is an infection of the endocardium, often including the valves, and is a complication of *S. aureus* bacteraemia (3). Infective endocarditis is associated with high morbidity and mortality (4, 5), and its development relies on interactions between *S. aureus* proteins and host proteins. The bacterial cell surface fibronectin (Fn)-binding^4^ protein A (FnBPA) has been implicated in this infection (6). The N-terminal region of FnBPA contains three subdomains N1–N3; N2N3 binds fibrinogen (Fg) and elastin (7, 8) (see Fig. 1*A*). The adjacent repetitive region of FnBPA contains multiple binding sites for Fn (Fig. 1*A*) (9, 10). Fn and Fg binding has been proposed to cooperate in infective endocarditis (6). FnBPA N2N3 has sequence homology with the N2N3 domains of the Fg-binding proteins ClfA (from *S. aureus*) (Fig. 1*B*) and SdrG (from *Staphylococcus epidermidis*); 24 and 22% of the residues are identical, respectively. ClfA has also been demonstrated to act as a virulence factor in infections such as septic arthritis (11) and experimental endocarditis (12). In ClfA (13) and SdrG (14), N2 and N3 both adopt an Ig-like fold; however, both proteins lack the C-terminal Fn-binding repeats found in FnBPA. The C terminus of SdrG contains two Cna_B (Pfam; PF05738) domains followed by a short serine-rich sequence, whereas ClfA lacks the Cna_B domains but has a longer serine-rich sequence (15). To date, there is no structural information for the N1 domain of Clfa, SdrG, or FnBPA.

Fg is a ∼340-kDa glycoprotein comprising a dimer of heterotrimers of chains Aα, Bβ, and γ (16). Fg is present in human blood at ∼2–4 g/liter (17) and, when converted to fibrin (through the action of thrombin), plays a key structural role in the clot (17). Binding sites for integrins (18) and growth factors (19) within Fg are involved in hemostasis and wound healing. ClfA (20) and FnBPA (21) both bind near the C terminus of the most abundant fibrinogen γ-chain isoform γA; SdrG binds the N terminus of the β chain (22). In binding to N2N3 of ClfA and SdrG, the different Fg peptides each form a β-strand conformation along the C-terminal strand of N3. Fg strand formation is parallel in ClfA binding to the γ-chain (23) and anti-parallel in SdrG binding (14) to the β-chain. In addition to strand formation, in the SdrG/Fg interaction, a C-terminal sequence beyond N3 binds back along N2 in a mechanism referred to as “dock, lock, latch,” with the N2-binding sequence being the “latch” (14). The ClfA-Fg interaction forms through a variation of this mechanism that includes latching strand formation along N2 (23).

Fn is a glycoprotein present in a soluble dimeric form (∼450 kDa) in human blood plasma (0.3–0.4 g/liter) (24) or in the form of insoluble fibrils in extracellular matrices (25, 26). Fibronectin plays important roles in cell migration during development and wound healing (27) and thrombosis (28). FnBPA targets the N-terminal domain (NTD) of Fn, which is composed of five F1 modules (^1–5^F1). The Fn-binding region of FnBPA comprises 363 residues and contains eleven homologous, intrinsically unstructured repeats (Fn-binding repeats (FnBRs)), FnBPA-1 to FnBPA-11 (8, 29, 30) (Fig. 1*A*). At least six FnBRs are able to bind with nanomolar affinity to the Fn NTD (31). Each FnBR/NTD interaction occurs via an unusual tandem β-zipper mechanism in which the FnBR forms an additional anti-parallel β-strand along the triple-stranded β-sheets of sequential F1 modules (32, 33).

Although structural details of the Fg-FnBPA interaction have been unknown to date, based on the ClfA/Fg peptide structure (23) and the FnBPA-1/NTD structure (33), the Fg- and Fn-binding sites on FnBPA are in close proximity (Fig. 1*B*), suggesting that cooperativity (positive or negative) might be involved in ternary complex formation. Here we solve the structure of N2N3 from FnBPA in its apo and Fg peptide-bound forms. We show that the latch strand is not required for Fg peptide or FgD (a protolytic fragment of Fg) binding and that only a minimal conformational change occurs in FnBPA on peptide binding. We show that although the Fg-binding site and the most N-terminal Fn-binding site on FnBPA are in close proximity, the residues involved are nonoverlapping. However, studies using intact Fn and Fg and their subdomains, and an FnBPA protein construct containing the adjacent Fg- and Fn-binding sites, provide evidence that Fg binding is sterically regulated by binding of Fn. This regulation is likely to have important implications for *S. aureus* interactions with host molecules *in vivo*.

## EXPERIMENTAL PROCEDURES

### 

#### 

##### Molecular Biology

Genes encoding the rFnBPA protein constructs (rFnBPA_(189–511)_, rFnBPA_(189–505)_, and AF1(rFnBPA_(189–550)_); Fig. 1*A*) were subcloned from a pQE-30 vector containing the cDNA of full-length FnBPA (GenBank^TM^ accession number J04151.1, *S. aureus* 8325-4 strain) into pET-YSBLIC-3C (34). Sequences were confirmed by in-house DNA sequencing.

##### Expression and Purification of rFnBPAs

rFnBPA constructs were expressed with an N-terminal His_6_ tag in *Escherichia coli* BL21 (DE3) Gold cells and purified using nickel affinity chromatography. The His tag was cleaved using HRV 3C protease, and after nickel affinity chromatography to remove the tag and uncleaved material, cleaved rFnBPAs were concentrated and further purified by size exclusion chromatography using a prepacked Superdex S75 16/65 HiLoad column (GE Healthcare). AF1 required additional anion exchange chromatography using HiTrap Q FF Sepharose (GE Healthcare) equilibrated in 20 mm Bis-Tris buffer, pH 6.2. The purity and molecular mass of final products were confirmed by SDS-PAGE and MS/ESI, respectively. Protein concentrations were determined from absorbance measurements at 280 nm.

##### Plasma Proteins and Peptides

Intact human plasma Fg (341 kDa, product no. 341576) and FgD (a monomeric ∼85-kDa proteolytic fragment of Fg containing the C-terminal regions of Aα, Bβ, and γ chains, product no. 341600) were purchased from Calbiochem-Merck-Millipore. Human plasma Fn (450 kDa, product no. F0895) and NTD (30-kDa proteolytic fragment of Fn, product no. F9911), which has been shown previously to bind FnBPA-1 (31), were purchased from Sigma-Aldrich. FgγC comprising the 17 C-terminal residues of the Fg γA chain (Ac-GEGQQHHLGGAKQAGDV-NH_2_) was purchased as a synthetic peptide from Severn Biotech Ltd. Fibronectin-free Fg was supplied by Enzyme Research Laboratories (Swansea, UK).

##### Isothermal Titration Calorimetry (ITC)

Experiments were performed using a MicroCal VP-ITC calorimeter (GE Healthcare) in PBS (140 mm NaCl, 2.7 mm KCl, 10 mm Na_2_PO_4_, 1.8 mm KH_2_PO_4_, pH 7.4) at 25 °C. Procedures were similar to those reported previously (35). Each titration started with one 2-μl injection followed by 27 × 10 μl injections at 0.5 μl/s using 6-min intervals. The stirring speed 307 rpm was used for all titrations except those involving Fg, where the stirring speed was increased to 321 rpm because of the higher viscosity of the Fg solution. Binding isotherms were fitted to a single-site binding model using nonlinear regression analysis in MicroCal-Origin 7.0 software.

##### Surface Plasmon Resonance (SPR)

Experiments were performed at 25 °C using a Biacore T100 system (GE Healthcare) upgraded to a T200 specification. Ligands in 10 mm sodium acetate (pH 5.5) were immobilized onto the experimental flow cell of a CM5 or C1 sensor chip (GE Healthcare) by amine coupling and subsequent blocking (36). The reference flow cell underwent identical but blank immobilization and blocking. Unless otherwise stated, low level immobilizations (50–150 response units) were used. The running buffer HBS-*p* + (10 mm HEPES, pH 7.4, 150 mm NaCl, 0.05% (v/v) polysorbate 20, GE Healthcare) was applied at 30 μl/min.

Analyte contact times were 60–300 s, dissociation times were 180–700 s, and stabilization times were 120–1500 s. Regenerations with low pH solutions were used only when needed and were optimized to minimize volume and harshness; pre- and postregeneration binding levels and curves were compared. Automated experiments were performed to measure dissociation constants (*K_d_* values) by kinetic or equilibrium methods, and binding/inhibition. Five prior start-up cycles ensured stable baselines. For *K_d_* determination, sensorgrams were measured for at least 10 sequential 2-fold analyte dilutions to cover the concentration range 0.1*K_d_*–10*K_d_*. Achievement of a steady-state response enabled *K_d_* determination from the equilibrium binding (37). Alternatively, kinetic data series were fitted to a Langmuir 1:1 binding model. Response differences between sample and reference cells were analyzed using Evaluation Software (GE Healthcare). For *K_d_* determination, an upper limit for analyte concentration for sensorgram analysis was determined to ensure reliable curve-fitting indicated by low χ^2^ values. In the binding/inhibition experiments, either AF1 or the NTD was immobilized; pure or mixed proteins with known concentrations were the analytes.

##### Crystallography

Diffracting crystals of rFnBPA_(189–505)_ grew in a Clear Strategy Screen II condition containing PEG 20000 (8%, w/v), PEG monomethyl ether 550 (8%, v/v), 0.2 m calcium acetate, with added 50 mm Tris-HCl, pH 7.4, 50 mm NaCl, at a protein concentration of 30 mg/ml using the sitting drop vapor diffusion method (38) combined with microseeding utilizing Seed Bead (Hampton Research). Clusters of protein crystals grew after 3 days at 18 °C. Clusters were transferred into cryo-protectant (reservoir solution with additional 10% (w/v) of PEG 20000 and 10% (v/v) of PEG monomethyl ether 550, from which a single diffracting crystal was broken off and vitrified in liquid nitrogen.

Diffracting crystals of the FnBPA_(189–505)_·FgγC peptide complex were grown in a Clear Strategy Screen II screen condition containing PEG 2000 monomethyl ether (25%, w/v), 0.2 m calcium acetate, with added 50 mm Tris-HCl, pH 7.4, 100 mm NaCl, isopropanol (10% (*v/v*)), at an FnBPA_(189–505)_ concentration of 25 mg/ml and a 20-fold molar excess of FgγC. Sitting drop vapor diffusion with the microseeding technique was set up as described above. Crystals, obtained after 48 h at 18 °C, were transferred into cryo-protectant (reservoir solution with additional PEG 2000 (5%, w/v) and vitrified in liquid nitrogen.

##### X-ray Data Collection, Structure Solution, and Refinement

Diffraction data for the rFnBPA_(189–505)_ and rFnBPA_(189–505)_·FgγC crystals were collected on the European Synchrotron Radiation Facility (Grenoble, France) Beamline ID29 and Diamond (UK) Beamline i04-1, respectively, at 100 K (Table 1). Diffraction data were indexed, integrated, and scaled using HKL2000 (39) and xia2 (40) for rFnBPA_(189–505)_ and the rFnBPA_(189–505)_·FgγC complex, respectively. The rFnBPA_(189–505)_ crystal diffracted to 2.19 Å, and the structure was solved using the molecular replacement pipeline BALBES (41) that chose a combination of the ClfA (Protein Data Bank code 1N67) and SdrG·Fg peptide complex (Protein Data Bank code 1r17) structures as a molecular replacement search model. After initial cycles of REFMAC (42), ARP/WARP (43, 44) was used to build a partial model. The model was completed by the iteration of manual building in COOT (45) and refinement by REFMAC (42). The final structure was validated using MolProbity (46). In the Ramachandran plot, 95.7% of residues are in the preferred region, 3.2% are in the allowed region, and 0.9% (three residues) are outliers. The rFnBPA_(189–505)_·FgγC crystal diffracted to 1.83 Å, and the structure was solved by molecular replacement using the structure of rFnBPA_(189–505)_ as a model. The refinement and validation was carried out as described above. The Ramachandran plot shows 96.5% of residues in the preferred region, 3.1% in the allowed region, and 0.3% (2 residues) as outliers. The atomic coordinates and structure factor amplitudes have been deposited in the Protein Data Bank under codes 4B5Z and 4B60.

**TABLE 1:** **Crystallographic data collection and refinement statistics for rFnBPA_(189–505)_ and the rFnBPA_(189–505)_·FgγC complex** Data for the highest resolution shell are shown in parentheses where applicable.

Parameters	rFnBPA_(189–505)_	rFnBPA_(189–505)_·FgγC
Beamline	ID29	I041
Space group	P2_1_2_1_2_1_	P1
Cell dimensions *a*, *b*, *c* (Å)	62.6, 75.2, 85.5	37.5, 59.1, 73.5
Cell dimensions α, β, γ (°)	90.0, 90.0, 90.0	91.8, 98.1, 97.9
Wavelength (Å)	0.9763	0.91731
Resolution (Å)	56.5–2.2	58.4–1.8
*R*_merge_	0.10 (0.52)	0.07 (0.36)
*I*/σ*I*	8.6 (6.0)	9.1 (2.0)
Completeness	99.2 (87.3)	93.6 (67.4)
Redundancy	6.7 (3.6)	2.2 (2.2)
No. of unique reflections	21,217	51,268
*R*_factor_	0.19	0.194
*R*_free_	0.24	0.247
Mean B-factor (Å^2^)	18.3	7.58
r.m.s.d. bond lengths (Å^2^)	0.02	0.02
r.m.s.d. bond angles (°)	1.89	1.95

##### S. aureus Fn Binding Experiments

*S. aureus* expressing FnBPA constructs with differing numbers of FnBRs were produced, and binding experiments to immobilized Fn were performed, as previously described (47). Briefly, human plasma fibronectin (Sigma) was diluted in PBS and immobilized onto the plastic wells of an ELISA plate by incubation at 4 °C for 16 h. Unoccupied binding sites were blocked with 3% bovine serum albumin before the addition of ∼10^8^ washed *S. aureus* cells expressing FnBPA constructs to each well. Experiments were done in the absence or presence of a range of concentrations of human fibrinogen. After incubation for 1 h at 37 °C, unbound bacteria were removed by three PBS washes. Adherent bacteria were quantified by crystal violet staining; bound dye was solubilized in 7% acetic acid, quantified by A_595_ measurements, and related to bacterial numbers by reference to standard plots (45).

##### Binding Site Occupancy Calculations

Relative occupancy of the Fg- and Fn-binding sites on AF1 was calculated by considering the binding as competitive. The apparent dissociation constant for Fg was determined according to the equation *K_d_*_Fg, app_ = *K_d_*_Fg_ (1 + [Fn]/*K_d_*_Fn_) (48). The average number of Fg molecules bound per AF1 molecule (α) was calculated using α_Fg_ = ([Fg]/(*K_d_*_app Fg_ + [Fg])). *K_d_*_Fn,app_ and α_Fn_ were calculated similarly.

## RESULTS

### 

#### 

##### Structure of rFnBPA N2N3

**FIGURE 2.**
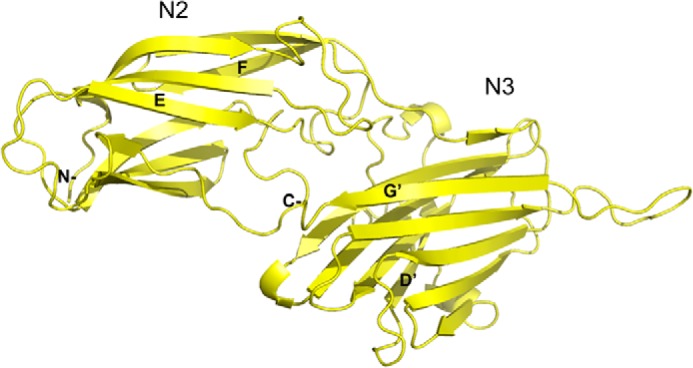
**Ribbon representation of the crystal structure of rFnBPA_(189–505)_·rFnBPA_(189–505)_ (Fig. 1*A*) is colored *yellow*.** The structures of the N2 and N3 domains are distorted β-barrels and resemble the topological arrangement of a variable IgG fold. β-Strands mentioned in the text are labeled; N and C termini are also labeled. The figure was generated using PyMOL (The PyMOL Molecular Graphics System, version 1.4.1).

Both rFnBPA_(189–511)_ (the N2N3 domains including the latch strand) and rFnBPA_(189–505)_ (without the majority of the equivalent strand-forming residues in the ClfA latch; Fig. 1) were used in crystallization trials. However, only rFnBPA_(189–505)_ produced diffracting crystals. The structure of rFnBPA_(189–505)_ comprises two distinct β-strand-dominated domains, N2 and N3, connected by an eight-residue linker (Fig. 2). The N-terminal N2 domain (residues 195–335) consists of nine β-strands arranged in a sandwich of four- and five-strand β-sheets. The N3 domain (residues 344–503) has a similar structure comprising two β-sheets, each formed by five β-strands. However, unlike N2, the two β-sheets within the N3 domain are linked by a short helix. The structures of N2 and N3 superimpose with a root mean square deviation (r.m.s.d.) of 3.17 Å. The structures of both domains resemble a distorted β-barrel rather than a typical β-sandwich. The N2 domain adopts the DE variant of a C-type IgG fold (DEv-IgG) identified in other proteins including ClfA (13). The asymmetric unit comprises a single copy of rFnBPA_(189–505)_; no density was observed for the N- and C-terminal residues (residues 189–194 and 504–505, respectively). The N2-N3 interface buries 1024 Å^2^ of surface area, and the main N2-N3 interdomain contacts originate from two regions: 1) interactions between two protruding loops that connect strands D′ and D1′ within the N3 domain and the E and F strands of the N2 domain and 2) interactions of the A′ strand of the N3 domain with a loop connecting the C and D strands in the N2 domain (Fig. 2).

##### The Latch Strand Stabilizes Fg Binding by Only ∼2.5-fold

**FIGURE 3.**
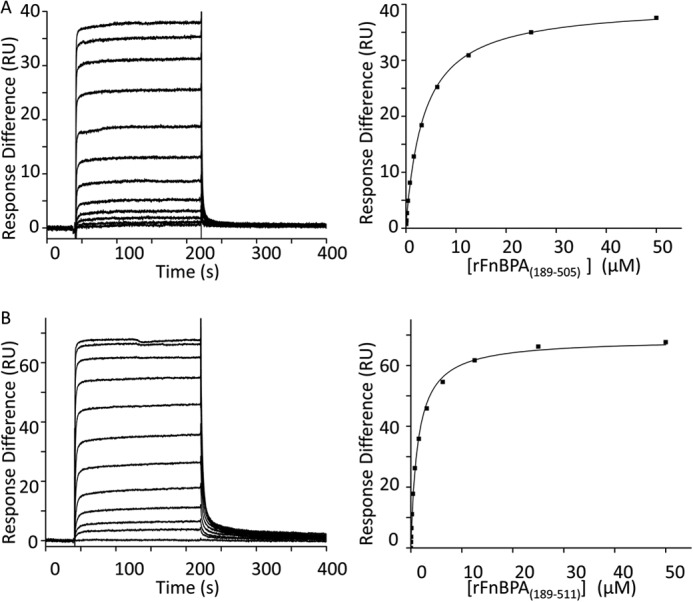
**SPR equilibrium analysis of the interactions between the analytes rFnBPA_(189–505)_ (*A*) and rFnBPA_(189–511)_ (*B*) with immobilized FgD.** rFnBPA_(189–505)_ and rFnBPA_(189–511)_ are recombinantly expressed regions of FnBPA (Fig. 1*A*) and FgD, which is immobilized on the chip, is a proteolytic fragment of Fg. *Left panels*, SPR sensorgrams for 2-fold dilutions of the analytes show that equilibrium is attained. *Right panels*, equilibrium response differences (*black squares*) and steady-state affinity fit (*black lines*). All data were obtained and processed as described under “Experimental Procedures.” The parameters derived from these experiments are shown in Table 2. *RU*, response units.

It was shown previously that the ClfA/Fg interactions are stabilized ∼10-fold by locking of the latch strand with a disulfide bond (23). To investigate potential stabilization provided by the FnBPA latch strand, the *K_d_* values for interactions between FgD and rFnBPA_(189–511)_ or rFnBPA_(189–505)_ (Fig. 1*A*) were measured using SPR (Fig. 3, *A* and *B*). FgD was immobilized on the surface of a chip and subsequently exposed to increasing concentrations of rFnBPA_(189–505)_ (Fig. 3*A*) orrFnBPA_(189–511)_ (Fig. 3*B*). *K_d_* values determined from equilibrium binding data were 3.7 ± 0.2 and 1.5 ± 0.1 μm for rFnBPA_(189–505)_ and rFnBPA_(189–511)_, respectively (Table 2). The similarity of these *K_d_* values demonstrates that the presence of the putative latch strand only stabilizes the FnBPA-FgD interaction by ∼2.5-fold. Hence, the rFnBPA_(189–505)_·FgγC peptide complex is a suitable minimal complex for crystallization.

**TABLE 2:** **Summary of kinetic and thermodynamic parameters for specific interactions** Dissociation constants (*K_d_* values) were determined by ITC or SPR or both techniques. Values for the enthalpy (Δ*H*) and entropy (Δ*S*) changes and stoichiometry (*n*) were also determined for those interactions studied by ITC. Errors are from the fit.

Interaction	*K_d_*	Δ*H*/kcal	ΔS/cal	*n*	Method
		*kcal mol*^−*1*^	*cal mol*^−*1*^ *K*^−*1*^		
rFnBPA_(189–505)_/FgD	3.7 ± 0.1 μm				SPR*^a^*
rFnBPA_(189–511)_/FgD	1.5 ± 0.1 μm				SPR*^a^*
AF1/Fg	1.1 ± 0.1 μm	−6.1 ± 0.0	6.7	2.11	ITC*^b^*
AF1/Fn	0.359 ± 0.001 nm				SPR*^b^*
AF1/FgD	1.5 ± 0.1 μm				SPR*^b^*
AF1/NTD	0.393 ± 0.002 nm				SPR*^b^*
AF1/NTD	0.7 ± 0.2 nm	−35.8 ± 0.3	−78.1	0.89	ITC*^b^*

*^a^* The data are shown in Fig. 3.

*^b^* The data are shown in Fig. 6.

##### Crystal Structure of the N2N3·FgγC Complex

**FIGURE 4.**
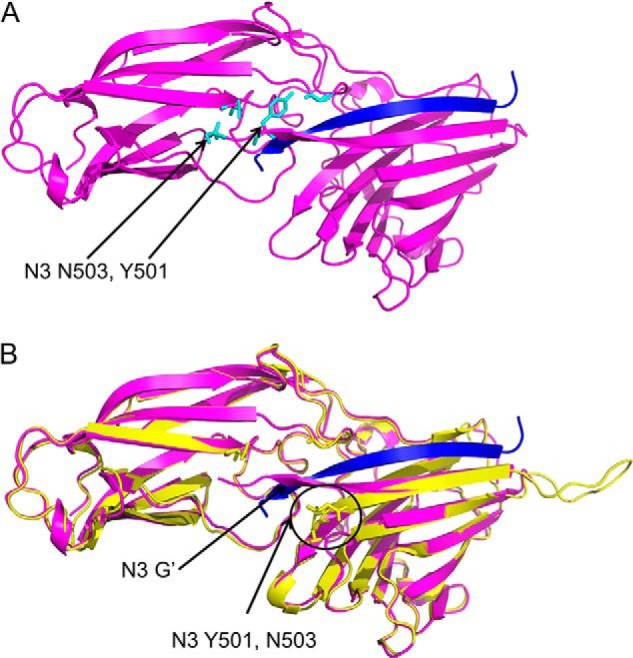
**Ribbon representations of the structure of the rFnBPA_(189–505)_·FgγC complex (*A*, the Fg peptide is shown in *blue*) and an overlay with the apo-structure (*B*, *yellow*).** The G strand of N3 and N3 residues mentioned in the text that adopt a different orientation on peptide binding are highlighted. The figures were generated and the structures aligned using PyMOL (The PyMOL Molecular Graphics System, version 1.4.1).

The rFnBPA_(189–505)_·FgγC complex was solved using x-ray crystallography (Fig. 4*A*). The asymmetric unit comprises two copies of the rFnBPA_(189–505)_·FgγC complex, referred to as A·C and B·D, which are almost identical: the r.m.s.d. between the A and B chains was 0.33 Å). Overall, the quality of the electron density map for the A·C complex was better than that for the B·D complex. Although a larger proportion of the molecule could be modeled into the A·C electron density, both maps contained clearly defined density corresponding to 14 and 12 amino acid residues of the full-length FgγC. Akin to the apo-protein structure, electron density corresponding to the N- and C-terminal residues (residues 189–194 and 504–505, respectively) of rFnBPA_(189–505)_ was absent. In contrast to the apo-protein structure, no electron density was detected within the N3 loop (residues 479–489) that connects the F′ and G′ strands. The FgγC peptide binding site is located in a cleft between N2 and N3. FgγC forms an additional β-strand parallel to the G′ strand in N3 in a β-zipper interaction (50). The r.m.s.d between the structures of free and FgγC-bound rFnBPA_(189–505)_ is 0.81 Å (Fig. 4*B*), confirming that binding of FgγC does not induce large conformational changes in the individual N2 and N3 domains or in their relative orientation. The translocation of the N3 G′ strand is the only significant change. In the apo-form, the G′ strand aligns anti-parallel to the neighboring F′ strand. In the FgγC-bound form, the G′ strand “wraps” around the C-terminal residues Gln-13, Ala-14, and Gly-15 of the peptide and interacts with the N2 domain. This was also observed in the ClfA peptide complex (23). Tyr-501 and Asn-503, which protrude away from the unbound rFnBPA_(189–505)_ structure, form hydrogen bonds with Gly-222 and Asn-304, respectively, in FgγC-bound rFnBPA_(189–505)_ (Fig. 4). The combined buried surface area within the rFnBPA_(189–505)_·FgγC A·C chain interface is 1880 Å^2^.

##### Can AF1 Bind Fg and Fn Simultaneously?

**FIGURE 5.**
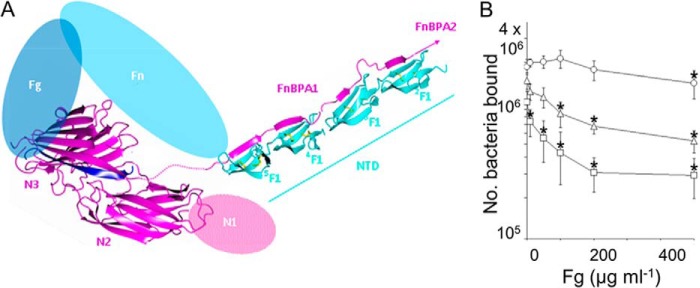
**The close proximity of Fn- and Fg-binding sites in *S. aureus* FnBPA suggests a mechanism for negative cooperativity.** *A*, ternary complex. Putative structure of the ternary complex formed by Fg·FnBPA·Fn. *Blue*, Fg; *magenta*, FnBPA; *cyan*, Fn. The model was prepared from three separate crystal structures: rFnBPA_(189–505)_·FgγC and two module pairs ^4^F1^5^F1 and ^2^F1^3^F1 in complex with synthetic peptides representing fragments of FnBPA-1 (Protein Data Bank codes 2RKY and 2RKZ) (33). Both module pairs are held together by disulfide bridges (*yellow*). Unmodeled regions of the three proteins are shown as *colored ovals* (not to scale). *B*, binding of *S. aureus* expressing various FnBPA constructs to Fn immobilized on plastic wells in the presence of increasing concentrations of Fg in solution. Values that were significantly different (*p* = < 0.05) from binding in the absence of soluble protein are indicated with *asterisks*. Experiments were repeated three times in duplicate. *Square*, FnBPA with only one FnBR (FnBPA-1); *triangle*, FnBPA with three FnBRs 1, 10, and 11; *circle*, intact FnBPA (*i.e.* with FnBRs 1–11).

The structure of the rFnBPA_(189–505)_·FgγC complex and of the previously determined structures of an FnBPA-1 (Fig. 1*A*) peptide in complex with ^4^F1^5^F1 from the NTD of Fn (33) demonstrate that, although nonoverlapping, the Fg-binding site on FnBPA is in close proximity to the most N-terminal Fn-binding site (FnBPA-1). Given that Fg and Fn are both large proteins, the question of whether binding of one protein to FnBPA affects the binding of the other (either though conformational change or steric exclusion) arises (Fig. 5*A*). First, the ability of *S. aureus* expressing FnBPA with differing numbers of FnBRs to bind to Fn in the presence of increasing concentrations of Fg was tested. In support of the existence of negative cooperativity between the adjacent Fg- and Fn-binding sites on FnBPA, addition of excess Fg disrupted binding of bacteria to a Fn-coated plate (Fig. 5*B*). Importantly, at the lowest concentrations of Fg, significant inhibition of binding to Fn was only observed when *S. aureus* expressing an FnBPA construct with only a single FnBR (FnBPA-1) was used. When more Fn-binding repeats were present, Fn binding plateaued at a higher level, suggesting, as might be expected, that only Fn binding to the FnBR adjacent to the Fg-binding site was inhibited by Fg. To investigate this potential negative cooperativity between Fg and Fn binding, AF1 (Fig. 1*A*) a recombinant FnBPA protein construct containing only the Fg (N2N3) binding site and the most N-terminal FnBR (FnBPA-1), was prepared. The first step was to measure the binding of the intact Fn and Fg and their subdomains to AF1.

**FIGURE 6.**
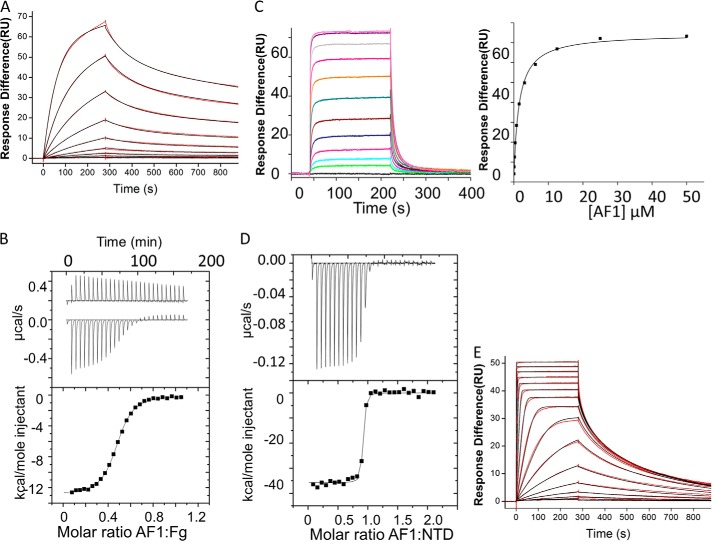
**Binding of AF1 to intact Fn and Fg and subdomains.** *A*, SPR sensorgram (*red*) and Langmuir kinetic binding fit (*black*) for 2-fold dilutions of dimeric human plasma Fn (5.2–0.01 nm) binding to AF1 (Fig. 1*A*) immobilized on a chip. *B*, heat generated on ligand injection (*upper panel*) and best fits of binding isotherms (*lower panel*) for the ITC titration of AF1 with Fg. *C*, SPR equilibrium binding analysis for AF1 (Fig. 1*A*) binding to FgD (immobilized), a proteolytic fragment of Fg. *D*, ITC experiment in which AF1 (Fig. 1*A*) is injected into NTD, a proteolytic fragment of Fn. *E*, SPR sensorgrams for the interaction of NTD with AF1 (immobilized). All data were obtained and processed as described under “Experimental Procedures.” Parameters derived from these experiments are shown in Table 2. *RU*, response units.

The *K_d_* values (Table 2) for the AF1-Fn (Fig. 6*A*; 0.36 ± 0.001 nm) and AF1/Fg interaction (Fig. 6*B*; 1.1 ± 0.1 μm) were measured using SPR kinetic analysis and ITC, respectively. The stoichiometry of 2:1 for the AF1/Fg interaction is consistent with the presence of two identical binding sites on intact dimeric Fg. The affinity of the AF1/FgD interaction (*K_d_* 1.5 ± 0.1 μm) was determined using SPR equilibrium analysis (Fig. 6*C*). The *K_d_* for the AF1/NTD interaction was determined using ITC (Fig. 6*D*) as 0.7 ± 0.2 nm. The steep transition of the binding curve resulted in a relatively high fit error (28%). Hence, this interaction was also measured using SPR kinetic analysis as 0.393 ± 0.002 nm (Fig. 6*E*) using increasing concentrations of the NTD as analyte and with AF1 immobilized onto a chip. Fig. 6 shows that both the Fg- and Fn-binding sites in AF1 are active. Because the *K_d_* values for the AF1 interactions with Fg and FgD and with Fn and NTD are virtually identical, there appears to be no steric occlusion of the binding sites within AF1 when individual proteins are binding. The physiological relevance of AF1 was assessed by a plasma pulldown assay, which demonstrated that AF1 also binds Fg and Fn under the conditions of human plasma (data not shown). However, this experiment does not reveal whether Fg and Fn can bind simultaneously to the same AF1 molecule.

**FIGURE 7.**
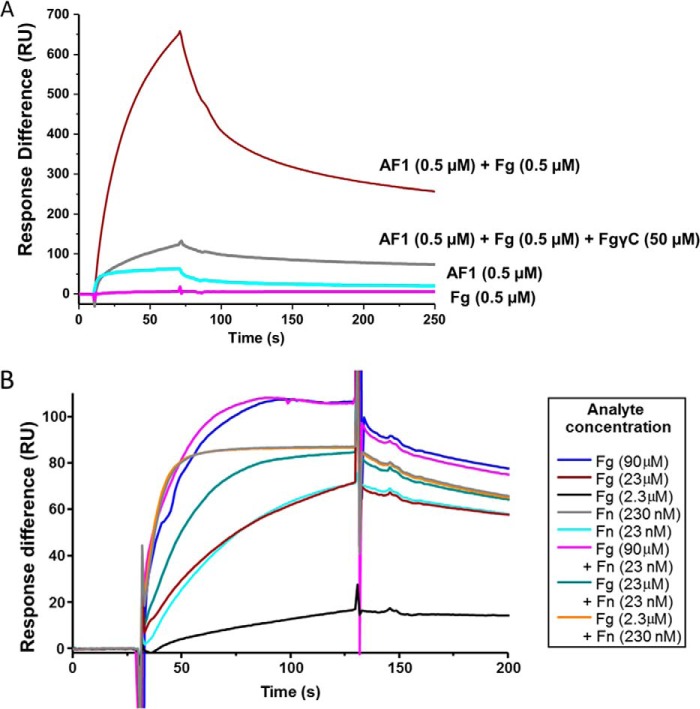
**Ternary complex formation and steric regulation.** *A*, representative SPR sensorgrams show responses from the interactions of NTD (a proteolytic fragment of Fn) immobilized on a C1 chip with AF1 (0.5 μm; Fig. 1*A*) in the presence or absence of an equimolar concentration of Fg. The effect of FgγC (50 μm) on the NTD·AF1·Fg ternary complex is also shown. The experiments shown were conducted sequentially on the same chip, and the order of experiments was as follows: 0.5 μm AF1 + 0.5 μm Fg; 0.5 μm AF1 + 0.5 μm Fg + 50 μm FgγC; 0.5 μm Fg; and 0.5 μm AF1. Regeneration was achieved with low pH. Repeats of 0.5 μm AF1 + 0.5 μm Fg were performed midway and at the end of the sequence of experiments to check binding after regeneration (data not shown). *B*, representative SPR sensorgrams from interactions of AF1 (Fig. 1*A*) with Fn and/or Fg. The experiments shown were conducted sequentially on the same CM5 chip with immobilized AF1. The order of experiments was as follows: 90 μm Fg; 90 μm Fg + 23 nm Fn; 23 nm Fn; 23 μm Fg + 23 nm Fn; 23 μm Fg + 230 nm Fn; 23 μm Fg; 2.3 μm Fg; and 230 nm Fn. Regeneration was achieved with low pH. Repeats of 90 μm Fg and 23 nm Fn were performed well separated in the sequence of experiments to demonstrate reproducibility of Fg and Fn binding after regeneration (data not shown). *RU*, response units.

Fig. 7*A* shows, for the first time, that a subdomain of Fn (NTD), AF1, and intact Fg can form a ternary complex. Exposure of an NTD-coated chip to an AF1·Fg complex (0.5 μm) generated a response exceeding 650 response units, whereas separate injections of AF1 (0.5 μm) and Fg (0.5 μm) only produced responses of 50 and 0 response units, respectively. The formation of the ternary complex was blocked by addition of FgγC. The results clearly demonstrate that AF1 can bind both Fg and NTD simultaneously to form a ternary complex.

The binding of Fg and intact Fn to AF1 was then investigated using SPR (Fig. 7*B*). An AF1-coated surface was exposed to a solution containing either Fg (2.3, 23, and 90 μm) or Fn (23 and 230 nm); binding was observed in all cases, showing that both Fg- and Fn-binding sites were active on the chip surface. Next, Fg and Fn were flowed over the chip together at concentrations of 2.3 μm and 230 nm, respectively. This reflects the 10:1 Fg:Fn ratio present in human plasma (49, 50). The binding trace was virtually identical to that observed for Fn only (at 230 nm), suggesting that no Fg interacts when the AF1-coated surface is saturated with Fn. Next, Fg (90 μm) was added to a solution of Fn (23 nm) and injected over the surface. Now the trace was virtually identical to that given upon injection of Fg only at 90 μm. This suggested that the molar excess of Fg saturated the AF1-coated surface and blocked the AF1-Fn interaction. Thus when intact proteins are used, it appears that no ternary complex is formed. Note that when subsaturating concentrations of Fn (23 nm) and Fg (23 μm) are combined, there is an additive response in the SPR trace that likely reflects binding of Fg and Fn to separate AF1 molecules on the chip.

##### Relative Occupancy of the Fg- and Fn-binding Sites of FnBPA in Plasma

When either Fg or Fn were present in large excess, SPR experiments suggested that the other ligand (Fn or Fg, respectively) could not bind (Fig. 7*B*) to AF1; that is, a ternary complex comprising AF1, Fn, and Fg was not observed. If only the affinities for the isolated binding sites in AF1 are considered, the reported concentrations of Fg (∼3 g/liter; 9 μm) and Fn (0.3 g/liter; 0.7 μm) in plasma would lead to relative occupancies of 0.89 and 1.0 for the Fg- and Fn-binding sites, respectively. However, Fig. 7*B* suggests that Fg and Fn compete for binding to AF1. It is possible to calculate a reduced “apparent *K_d_*” for binding of one protein to FnBPA in the presence of a specific concentration of an inhibitor. When the concentration of the two proteins in the plasma is taken into account, the relative occupancies of the Fg- and Fn-binding sites at equilibrium are predicted to be 0.004 and 0.99, respectively. This suggests that the Fg-binding site on FnBPA is predominantly unoccupied during *S. aureus* bacteremia.

## DISCUSSION

FnBPA was previously identified as a virulence factor in *S. aureus* infective endocarditis, with the Fg- and Fn-binding sites (21) synergistically promoting infection (6, 51). This work set out to characterize the FnBPA Fg-binding domain and its interaction with Fg and to test whether Fg and Fn can bind FnBPA simultaneously. Although the FnBPA/Fn interaction has been the subject of extensive structural characterization (31, 33, 52), no experimentally determined structure of FnBPA bound to its target site in Fg has previously been reported.

Previous studies of Fg binding to the bacterial proteins SdrG and ClfA revealed a mechanism of binding called dock, lock, latch (14). In SdrG, on binding of the Fg peptide the latch (a sequence at the C terminus of N3) forms a stabilizing strand/strand interaction along a β-strand in N2. In ClfA, the role of the latch is less clear (Protein Data Bank code 2VR3 (23)) because the deposited Fg peptide/ClfA structure contains an engineered disulfide bond between the latch and N2. In the apo-structure the latch sequence was either significantly truncated in the modeled structure, suggesting disorder (SdrG; Protein Data Bank code 2R19 (14)), or was stabilized by a short strand/strand interaction along N3 (ClfA; Protein Data Bank code 1N67 (13)). Sequence similarity between ClfA and FnBPA, the demonstration that the target site in Fg is the same, and that the affinity of the interaction is similar suggest both a similar mechanism of Fg binding for the two *S. aureus* proteins and a potentially puzzling level of redundancy.

In the present study, only a construct (FnBPA_(189–505)_) lacking the majority of the latch strand residues formed well diffracting crystals, and the affinity of the interaction of FgD with N2N3-containing constructs was reduced only ∼2.5-fold by this truncation (Fig. 3). Further truncation (to FnBPA residue 498) has been previously shown to disrupt Fg and elastin binding (8). However, inspection of both the previously determined ClfA/Fg peptide structures and the FnBPA_(189–505)_·FgγC complex determined here show that this truncation would remove intradomain interactions within N3 rather than only latching residues.

The structures of N2N3 (without latch) in isolation and in complex with FgγC revealed, as expected, similarities in the apo-structures and in the mechanism of FgγC binding between FnPBA and ClfA. Both N2 and N3 adopt Ig-like folds; N2 adopted the DEv-IgG variant described previously (13). The structure of the complex revealed parallel β-zipper binding by the peptide along the G′ strand of N3 (as in ClfA) and within a cleft formed predominantly by residues with hydrophobic side chains, stretching between the N2 and N3 domains. Previous studies (53, 54) suggested the C terminus of the Fg γ chain is intrinsically disordered. Thus it is likely to undergo a disorder to order transition upon binding to FnBPA. It fits into the cleft, causing the N3-G′ strand to translocate and wrap around the Fg peptide C terminus, establishing a relatively large interface.

There are also important differences in the binding mechanism compared with ClfA. Clearly, the latch β-strand residues are absent in the FnBPA_(189–505)_·FgγC structure. Rather, the data suggest that only the first few residues of the putative latch region (Tyr-501 and Asn-503) might be required for Fg binding. In a recent structural analysis of N2N3 from ClfB binding to a peptide from the Fg α-chain, a latching strand interaction did not appear to be required (55). This truncation of the mechanism to dock and lock was suggested to occur because the ClfB-binding site in the α-chain of Fg is midchain rather than at the C terminus. However, the results presented here suggest that latching is not always required even for C-terminal Fg peptide binding. In addition, the relative orientations of the N2 and N3 domains in FnBPA_(189–505)_ do not change significantly upon FgγC binding, unlike in binding of ClfA to FgγC.

FnBPA (containing adjacent Fg- and Fn-binding sites) represents an unusual opportunity to characterize formation of a ternary complex. Previous structural studies of FnBPA-1 in complex with the Fn F1 module pair ^4^F1^5^F1 and the structure of N2N3 in complex with an Fg peptide presented here clearly show that the adjacent binding sites for Fg and Fn in intact FnBPA are in close proximity but do not overlap. In particular, the lack of the requirement for latching strand residues in the rFnBPA_(189–505)_/FgD interactions (Table 2) suggested larger potential separation in the primary sequence between residues in FnBPA directly involved in binding of Fg and those affected by binding of Fn to the most N-terminal FnBR (FnBPA-1). However, given that both the rFnBPA_(189–505)_/FgγC and FnBPA-1/^4^F1^5^F1 interactions involve strand formation, the potential for positive cooperativity through latch strand stabilization adjacent to the strand-forming residues of FnBPA-1 exists (Fig. 5*A*). For example, positive cooperativity was observed previously in NTD binding to adjacent FnBRs in a streptococcal protein (56). Alternatively, steric regulation of binding to the adjacent sites (because both intact Fg and intact Fn are large proteins; Fig. 5*A*) might occur. Fig. 5*B* provides the first evidence for steric regulation of the adjacent binding sites when FnBPA is expressed on the surface of *S. aureus*.

Investigations at the molecular level show that the adjacent Fg- and Fn-binding sites of FnBPA (when expressed recombinantly as AF1) can form a ternary complex with Fg and the NTD subdomain of Fn (Fig. 7*A*). However, in Fig. 7*B* in which intact Fg *and* Fn were used, ternary complex formation was not observed. These two results together argue in favor of negative cooperativity arising though steric exclusion. The linear, and nonoverlapping, nature of the Fg- and Fn-binding sites in AF1 suggests that this result can be extrapolated to intact FnBPA and provides an explanation for the inhibition observed in Fig. 5*B*.

Although Fg is at a 10-fold higher concentration than Fn in the plasma, occupancy analysis predicts that Fn binding predominates because of the higher affinity of the interaction. This makes sense because FnBPA, although having only one Fg-binding site, has multiple Fn-binding sites (31), so inhibition of binding of Fn (by Fg) to the most N-terminal site would have little overall affect because Fn could (presumably) bind at the other sites unaffected by Fg binding.

Intriguingly, the binding of ClfA to Fg has been shown previously also to be negatively regulated, in this case by Ca^2+^-binding (57). The Ca^2+^-binding site is not conserved in the N2N3 domains of FnBPA, so it is interesting that a different mechanism of negative regulation seems to present. The importance of Fg binding (by either FnBPA or ClfA) for the initiation of infection in infective endocarditis has been clearly demonstrated (6, 51). How might regulation of the Fg-binding site though steric exclusion (or Ca^2+^ binding) aid initiation of infection? One possibility, suggested previously by O'Connell *et al.* (57) is that negative regulation of Fg binding might result in targeting of *S. aureus* to solid phase fibrinogen or fibrin clots. The C terminus of the γ chain of Fg is not only present in soluble Fg but also in cross-linked fibrin, and here it will have a higher local concentration and may thus be able to compete with Fn binding. *S. aureus* has previously been shown to bind more effectively to fibrin than fibrinogen (58). In the case of FnBPA where steric regulation appears to be operating, the accessibility of the binding site is also likely to play a role. Lastly, FnBPA also binds elastin (8) at a site that overlaps with the Fg peptide-binding site. Elastin binding to FnBPA was inhibited at lower concentrations of Fg peptide than Fg binding to FnBPA (8). Thus it is also possible that maintenance of a free binding site in N2N3 results in targeting of bacteria to elastin-rich tissues such as blood vessel walls during the initiation of infection.

## Footnotes

4 The abbreviations used are:

Fn: fibronectin
Fg: fibrinogen
FnBPA: fibronectin-binding protein A
rFnBPA: recombinant fragment of FnBPA
NTD: N-terminal domain of fibronectin (proteolytic fragment)
FgD: proteolytic fragment of Fg
FnBR: fibronectin (NTD)-binding repeat, FnBPA-1-11
FgγC: synthetic peptide comprising 17 C-terminal residues of the Fg γA chain
AF1: recombinant FnBPA protein construct comprising the adjacent Fg- and Fn-binding sites in FnBPA
r.m.s.d.: root mean square deviation
ITC: isothermal titration calorimetry
SPR: surface plasmon resonance.
